# A Mokken analysis of the literacy in musculoskeletal problems questionnaire

**DOI:** 10.1186/s12955-017-0826-2

**Published:** 2017-12-21

**Authors:** Brett Vaughan, Jane Mulcahy, Amy Coffey, Laura Addinsall, Stephanie Ryan, Kylie Fitzgerald

**Affiliations:** 1 College of Health & Biomedicine, Victoria University, Melbourne, Australia; 2 Institute for Sport, Exercise and Active Living, Victoria University, Melbourne, Australia

**Keywords:** Health literacy, Musculoskeletal, osteopathic medicine, Osteopathy, Manual therapy, Evaluation

## Abstract

**Background:**

Limited health literacy is known to impact on medication adherence, hospital readmission and potentially poorer health outcomes. The literature on the health literacy of those with musculoskeletal conditions suggests greater functional limitations and increased pain levels. There are a number of measures of health literacy. One that specifically relates to musculoskeletal complaints is the Literacy in Musculoskeletal Problems (LiMP) questionnaire. The LiMP contains 9 multiple choice items that cover anatomy, musculoskeletal conditions and the diagnosis of musculoskeletal complaints. The aim of the study was to evaluate the dimensionality and internal structure of the LiMP in patients attending for osteopathy care at a student-led clinic, as a potential measure of musculoskeletal health literacy.

**Method:**

Three hundred and sixty-one (*n* = 361) new patients attending the Victoria University Osteopathy Clinic completed the LiMP and a demographic and health information questionnaire prior to their initial consultation. Mokken scale analysis, a nonparametric item response theory approach, was used to evaluate the dimensionality and structure of the LiMP in this population, to ascertain whether the questionnaire was measuring a single latent construct – musculoskeletal health literacy. McDonald’s omega and Cronbach’s alpha were calculated as the reliability estimations. The relationship between the LiMP and a single item screen of health literacy was also undertaken.

**Results:**

The 9 items on the LiMP did not form a Mokken scale and the reliability estimations were below an acceptable level (alpha and omega <0.45). LiMP items 5 and 8 were more likely to be answered correctly by those with higher health literacy (*p* < 0.05), however the effect sizes were small (<0.20).

**Conclusion:**

Calculation of a total score for the LiMP, as advocated by the original authors, is not supported based on data in the present study. Further research is required to explore the relationship of the LiMP items to demographic and clinical data, and to other broader measures of health literacy. Further research may also develop a health literacy measure that is specific to patients seeking manual therapy care for musculoskeletal complaints.

## Introduction

The burden of musculoskeletal disorders in society is substantial [1–3] and one strategy to reduce this burden may be addressing health literacy (HL). Although HL has received considerable attention in the health professions literature [4, 5] there are few examples in the manual therapies. A systematic review by Loke et al. [6] suggests between 7 and 42% of those with chronic musculoskeletal complaints demonstrate low health literacy, with estimates varying depending upon the population, and type of measure utilised [7]. Vaughan et al. [8] evaluated the HL of patient’s attending an osteopathy, on-campus student-led clinic using a single item screening question, which identified 10% of patients attending this clinic had below adequate HL. Those patients who did not speak English at home, those with lower education levels, and those who were less satisfied with their life demonstrated lower levels of HL.

Although a range of measures exist to evaluate HL [9–11] they can be time-consuming to complete, or require administration by a research or practice assistant. Further, there are no specific questionnaires that exist in the literature to evaluate the HL of patient’s attending for manual therapy care. Rosenbaum et al. [12] state that “…musculoskeletal health literacy is thought to require a more sophisticated set of skills than those deemed crucial for general health literacy” (p. 608). This assertion is possibly supported by Briggs et al. [13] who identified that a cohort of chronic low back pain (LBP) patients had “...difficulties in seeking, understanding and utilising LBP information” (p. 275) despite having adequate HL scores using the Short Test of Functional Health Literacy in Adults.

The Literacy in Musculoskeletal Problems (LiMP) questionnaire was developed to evaluate the “...competencies that are integral to making informed decisions regarding musculoskeletal health” (p. 610) [12]. The LiMP consists of 9 items across three domains: anatomy and terminology, musculoskeletal conditions, and diagnosis and treatment. The questionnaire takes 5 min to complete. Patients are asked to select a response for each item (5 response options for items 1–8 and 4 response options for item 9) and only one response is permissible. The response to each item is scored as correct (1) or incorrect (0) and the score totalled for a possible score of 9. LiMP scores below 6 are reported to indicate limited musculoskeletal HL and “…scores of ≥6 can effectively rule out both limited musculoskeletal literacy and general health literacy” (p. 191) [14]. The LiMP has been used to evaluate the musculoskeletal HL of those with carpal tunnel syndrome [15], foot and ankle complaints [16], those presenting to an emergency department [14, 17] and an orthopaedic outpatients clinic [18]. It has a Flesch-Kincaid grade level of 4.2 (able to be read by those with a 4th grade education or above) [17].

There is limited information about the dimensionality and internal structure of the LiMP, both a part of the argument for the validity of the questionnaire. Initial analysis identified a moderate positive correlation (0.41) between Newest Vital Sign [19] scores and the LiMP, and a Cronbach’s α of 0.59 [17]. Beyond Cronbach’s alpha, there is limited information to provide support for the internal structure of the LiMP. The aim of the present study is to evaluate the dimensionality and internal structure of the LiMP to ascertain if it measures a single latent construct (musculoskeletal health literacy) in a patient population attending a student-led osteopathy clinic for the management of musculoskeletal complaints.

## Methods

This study forms part of a larger investigation into the demographics and health screening of patient’s attending the VU Osteopathy Clinic, a student-led osteopathy teaching clinic at Victoria University (VU) in Melbourne, Australia. The VU Human Research Ethics Committee approved the study (HRE15–005) and the study was conducted between March 1 and June 30, 2016.

### Participants

All new patients attending the VU Osteopathy Clinic were asked to complete a demographic and health screening questionnaire devised by the researchers, along with the LiMP. Patients completed the questionnaires in the reception area of the clinic prior to their initial osteopathy treatment, however completion was not required to receive care. The demographic data, health screening data and LiMP were de-identified by one of the authors (BV) prior to data entry.

### Data analysis

Data were entered into SPSS (IBM Corp, USA) then exported to *R* [20] for analysis. Descriptive data analysis was undertaken in the *psych* package [21]. LiMP scores were entered as polytomous data and missing data for the LiMP were imputed using a two-way imputation [22] from the *TestDataImputation* package [23]. After imputation, scores were recoded to dichotomous to reflect a correct (1) or incorrect (0) response for the item. The focus of the analysis presented here is the psychometric properties of the LiMP using Mokken scale analysis (MSA). MSA is a non-parametric item response theory approach used to evaluate dimensionality and the internal structure of a scale or questionnaire [24, 25]. Readers are directed to other authors for a more in-depth review of MSA [25–28]. The *mokken* package [27] was used to perform a Mokken scale analysis (MSA) following the procedures and steps described by Stochl et al. [26] and Van der Ark [27], and outlined in Table 1. The mathematical basis for the scalability coefficients is found in Stochl et al. [26]

**Table 1:** Steps in the Mokken scale analysis for dichotomous items outlined by Stochl et al. [26]

Step 1a	Dimensionality assessment (using the *coefH* function in the *mokken* package in *R*). Produces scalability coefficient for all items (*H*), item pairs (*Hij*), and individual items (*Hi*)). Values for each should be between 0 and 1.
Step 1b	Formation of Mokken scales (using the automated item search procedure or *aisp* function in the *mokken* package in *R*). Items form a coherent scale.
Step 2	Assessment of monotonicity (using the *check.monotonicity* function in the *mokken* package in *R*). Score for an individual item should monotonically increase for an increase in the value associated with the latent trait).
Step 3	Assessment of invariant item ordering (IIO) and non-intersection (using the *check.iio* function in the *mokken* package in R). The item characteristic curves for a score of 0 or should not cross as the latent trait changes. IIO is where the ‘difficulty’ of the item does not change as the value associated with the latent trait changes.

McDonald’s omega [29] was the selected reliability estimation method to evaluate internal structure, and was calculated using the *userfriendlyscience* package [30]. This estimation was selected as it does not require tau-equivalence. Both McDonald’s omega total (ωt) and omega hierarchal (ωh) were calculated with values over 0.7 being acceptable [31]. Omega total (ωt) is the “…estimate of the proportion of total common variance in the test” (pp. 152) [32] whereas ωh is “…the extent to which a scale score estimates a latent variable common to all items” (pp. 5) [33]. Cronbach’s alpha was also calculated for comparison with the Rosenbaum et al. [17] study using the *userfriendlyscience* package [30]. The data likely violates the tau-equivalence assumption to calculate alpha, thereby limiting the ability to meaningfully interpret this value [33]. Further, alpha calculations often “…leads to overestimates of reliability and undercorrections for biases due to measurement error” (pp. 206) [34]. These issues with α support the use of ω as the reliability estimation method in the present study. The Mann-Whitney U test was used to evaluate whether the total LiMP score was significantly influenced by health literacy as measured by the single item “*How confident are you completing medical forms*?” [35]. Alpha was set at 0.05 and the effect size (*r*) was calculated and interpreted according to Yatani [36].

## Results

**Fig. 1:**
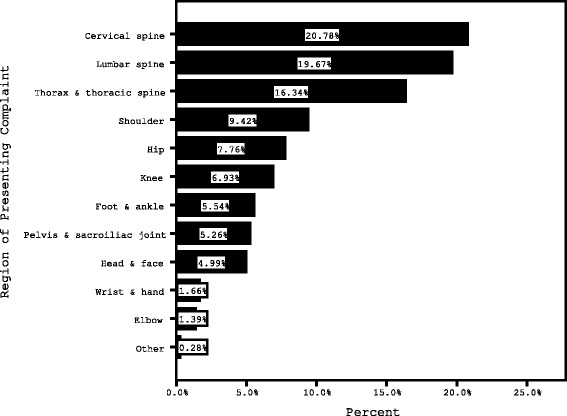
Region of patient complaints presenting to the VU Osteopathy Clinic

Four hundred and forty (*N* = 440) new patients attended the student-led clinic between March and June 2016, with demographic and health information data available for 414 patients. The 26 patients whose data was not available for analysis included those under the age of 18 years (*n* = 2) and patients who indicated they did not consent for their data to be used in the study (*n* = 24). Fifty-four (*n* = 53, 13%) responses were removed as the patient did not complete the LiMP leaving 361 responses to analyse. Selected demographic data and the body region of the presenting complaint are presented in Table 2 and Fig. 1 respectively. Percentages for correct responses to each of the 9 LiMP items are presented in Table 3.

**Table 2:** Demographic data for patients participating in the study before and after removal of those patients who did not complete the Literacy in Musculoskeletal Problems (LiMP) questionnaire

	All 414 available data sets	After removal of those patients who did not complete the LiMP
Gender		
Male	158 (38.2%)	142 (39.3%)
Female	253 (61.1%)	219 (60.7%)
Missing	3 (0.7%)	
Age		
Mean (±SD) years	32.6 (±12.3)	32.5 (±12.3)
Range	18–77 years	18–77 years
Stage		
Acute	229 (55.3%)	206 (57.1%)
Chronic	182 (44.0%)	155 (42.9%)
Missing	3 (0.7%)	
Clinic		
Melbourne city	351 (84.8%)	313 (86.7%)
St Albans	60 (14.5%)	48 (13.3%)
Missing	3 (0.7%)	
Highest education level attended		
High school (not completed)	15 (3.6%)	12 (3.3%)
High school (completed)	44 (10.6%)	35 (9.7%)
Trade or vocational education	55 (13.3%)	53 (14.7%)
University	293 (70.8%)	259 (71.7%)
Missing	7 (1.7%)	2 (0.6%)
Completing medical forms		
Not at all confident	3 (0.7%)	1 (0.3%)
A little confident	5 (1.2%)	5 (1.4%)
Somewhat confident	28 (6.8%)	21 (5.8%)
Quite confident	133 (32.1%)	122 (33.8%)
Extremely confident	228 (55.1%)	205 (56.8%)
Missing	17 (4.1%)	7 (1.9)
General health (median, range)	3 (1–5)	3 (1–5)
Missing	8 (1.9%)	2 (0.6%)
Satisfaction with life (median, range)	4 (0–5)	4 (0–5)
Missing	9 (2.2%)	3 (0.8%)

**Table 3:** Literacy in Musculoskeletal Problems questions with percentage of correct responses

Question	Percentage correct
1. A “fractured” bone is	47.4%
2. All of the following facts about X-rays are true EXCEPT:	26.6%
3. What is the name of the bone in your thigh?	67.9%
4. An Orthopaedic Surgeon is	68.4%
5. What is sciatica?	57.6%
6. The knee is a	78.9%
7. Arthritis is	49.3%
8. How does Rheumatoid Arthritis (RA) differ from Osteoarthritis (OA)?	41.8%
9. If you break your wrist, what might you doctor give you to help you heal?	33.5%

Initial scalability of the 9-item LiMP suggested that it was a ‘weak’ scale (0.14 ± 0.02) that is, the items together do not measure a single latent construct. Individual item scalability was also low (*Hi* < 0.24). A negative *Hij* coefficient was identified for item 9 with all other items. The *aisp* function was subsequently used to identify possible Mokken scales. With the lowerbound set at 0.3, the minimum for a Mokken scale, two potential scales were identified: one being items 3, 5, and 8; the second being items 6 and 7. A lowerbound value of 0.40 suggested items 3 and 8 only formed a Mokken scale. Setting the lowerbound value to 0.5 suggested that none of the 9 items would create a Mokken scale. McDonald’s ωt (0.52, 95%CI 0.45–0.59) and ωh (0.27), and Cronbach’s alpha (0.49, 95%CI 0.41–0.57) for the 9-item scale were all below acceptable values.

As a total score for the LiMP is not valid each individual item was evaluated to ascertain if the correct/incorrect answer for the individual LiMP item was significantly based on the single item health literacy screen “How confident are you completing medical forms?” [35]. Those patients with higher health literacy were more likely to answer item 5 *What is sciatica?* (*p* = 0.011, *r* = 0.13) and item 8 *How does Rheumatoid Arthritis (RA) differ from Osteoarthritis (OA)* (*p* < 0.01, *r* = 0.18) correctly by, although the effect sizes were small.

## Discussion & conclusion

The present study sought to evaluate the dimensionality and internal structure of the LiMP in a patient population attending a student-led osteopathy clinic for the management of musculoskeletal complaints. Although Rosenbaum et al. [17] suggest “the LiMP is a valid tool for specifically assessing musculoskeletal health literacy…” (p. 405) the data presented here do not support this conclusion. Mokken scale analysis was used to evaluate the dimensionality of the LiMP when completed by patients presenting with a range of musculoskeletal complaints. This statistical approach provides evidence that the 9 LiMP items do not form a single scale. Rosenbaum et al. [17] report that whilst “…the LiMP does ultimately assess musculoskeletal literacy, it does so through an evaluation of several themes that cumulatively, not independently, determine one’s musculoskeletal literacy” (p. 404). This statement suggests that a total score for the LiMP is appropriate as a measure of a patients’ musculoskeletal health literacy. The calculation of a total score for the LiMP is not supported by the MSA or the McDonald’s ωh value. This raises questions about the use of the LiMP total score as a musculoskeletal HL screening tool.

Although one, or possibly two, Mokken scale(s) were identified, the fact these scales only contain two items severely limits their utility as a measurement tool. The value of the LiMP may be in the relationship of individual items to health screening data, rather than the creation of a total score for the questionnaire. Item 5 *What is sciatica?* and item 8 *How does Rheumatoid Arthritis (RA) differ from Osteoarthritis (OA)* were more likely to be answered correctly by those with higher self-reported health literacy. It is not clear why these two items only would be significantly different albeit with small effect sizes. Both items evaluate knowledge of musculoskeletal conditions as do item 1 *A “fractured” bone is* and item 7 *Arthritis is* therefore the difference may not be associated with knowledge level. Further research is required to ascertain if the significant difference is related to the population in the current study (possible given the small effect size), an issue with the structure of the LiMP items, or is a reflection of the patients’ knowledge of musculoskeletal complaints.

There are a number of limitations in the present study including convenience sample of patients attending the VU Osteopathy Clinic during the study period, and that patients were attending specifically for osteopathy care. Whether the results of the present study are generalizable to other professions managing musculoskeletal complaints, or attending a private practice setting, requires further investigation. Researchers are encouraged to utilise the LiMP and evaluate the dimensionality and internal structure in their clinical setting, to determine if these properties are consistent in other patient populations. Further research into the LiMP could also utilise parametric item response theory approaches such as Rasch analysis whereby stricter assumptions are placed on the data. The current research suggests that the LiMP in its current format is not appropriate for use in a population seeking osteopathy care. Use of a questionnaire that evaluates the health literacy of patients specifically seeking care for a musculoskeletal complaint, where the care is provided by a manual therapist may be of value. Continued efforts to evaluate the health literacy of those patients attending for the care of a musculoskeletal complaint is warranted given the substantial, and increasing, burden of musculoskeletal complaints worldwide.
